# Illegal products containing selective androgen receptor modulators purchased online from Italy: health risks for consumers

**DOI:** 10.1093/sexmed/qfae018

**Published:** 2024-03-27

**Authors:** Maria Cristina Gaudiano, Federica Aureli, Livia Manna, Anna Borioni, Alessandro Maccelli, Mariangela Raimondo, Donato De Giorgi, Monica Bartolomei

**Affiliations:** Centro nazionale per il controllo e la valutazione dei farmaci, Istituto Superiore di Sanità, Rome, 00161, Italy; Centro nazionale per il controllo e la valutazione dei farmaci, Istituto Superiore di Sanità, Rome, 00161, Italy; Centro nazionale per il controllo e la valutazione dei farmaci, Istituto Superiore di Sanità, Rome, 00161, Italy; Centro nazionale per il controllo e la valutazione dei farmaci, Istituto Superiore di Sanità, Rome, 00161, Italy; Centro nazionale per il controllo e la valutazione dei farmaci, Istituto Superiore di Sanità, Rome, 00161, Italy; Centro nazionale per il controllo e la valutazione dei farmaci, Istituto Superiore di Sanità, Rome, 00161, Italy; Centro nazionale per il controllo e la valutazione dei farmaci, Istituto Superiore di Sanità, Rome, 00161, Italy; Centro nazionale per il controllo e la valutazione dei farmaci, Istituto Superiore di Sanità, Rome, 00161, Italy

**Keywords:** SARMs, illegal medicines, 19F NMR, mass spectrometry, retail websites

## Abstract

**Background:**

Selective androgen receptor modulators (SARMs) are small synthetic drug molecules that are still not approved as medicine in Europe or the United States but are sold on illegal websites to improve sport performance, particularly bodybuilding.

**Aim:**

To address the quality issues of illegal SARM products and their increasing diffusion in Italy with their potential health risks for consumers.

**Methods:**

Web-based tools were used to investigate retail websites, trending searches, and information exchange via social media. Thirteen SARM products, purchased on retail websites accessible from Italy, were subject to visual inspection and chemical analysis by mass spectrometry and quantitative nuclear magnetic resonance.

**Outcomes:**

The primary outcome was demonstration of additional health risks due to the illicit presence of other active ingredients, contamination, and misdosage in SARM products sold on the internet. The secondary outcome was to show the increasing trend of interest in Italy for these products.

**Results:**

Most websites reported misleading information; specifically, the statement “for research only” was reported notwithstanding indications on dosage and training phases. The trending search showed that interest toward SARMs increased in Italy in the last years. The use of these products is clearly encouraged by the emerging phenomenon of “broscience” as revealed in socials. Visual inspection evidenced nonconform labeling. Qualitative analysis confirmed the presence of the stated SARM in about 70% of samples. In 23% of samples, the expected SARM was not detected but a different one instead, and in 1 sample, no SARMs were detected. Other undeclared pharmaceutical substances (tamoxifen, clomifene, testosterone, epimethandienone, tadalafil) were measured in 30% of samples. The copresence of >1 active substance was observed in >60% of samples. Quantitative nuclear magnetic resonance data showed nonuniform content ranging from 30% to 90% of the label claim.

**Clinical Implications:**

The use of SARMs, in the presence of unexpected life-threatening reactions in persons using the products to increase sport performance, should be assessed.

**Strengths and Limitations:**

This investigation involved an integrated approach to study SARM products and related sociologic aspects. The main shortcomings are the limited number of samples and retail websites in the clear web investigated.

**Conclusion:**

SARMs sold online as food supplement–like products represent a health hazard due to the presence of unapproved and undeclared active substances. The presence of contaminants clearly indicates the absence of good manufacturing practices in the production, which increases the health risks.

## Introduction

Selective androgen receptor modulators (SARMs) are small synthetic drug molecules presenting a plethora of chemical core structures (arylpropionamides, bicyclic hydantoins, quinolines, tetrahydroquinolines, etc) explored to modulate the function of the androgen receptor, with either receptor agonism or antagonism activity.^1^^,^^2^ The androgen receptor is involved in male sexual development and influences bone density, muscle mass, hematopoiesis, and metabolism. After the success of selective estrogen receptor modulators (eg, tamoxifen and clomifene) in the late 1990s, many public and private laboratories have taken steps to study molecules acting on the androgen receptor in view of possible medical applications minimizing the side effects in nontarget tissues.^3^^,^^4^ Specifically, SARMs were studied for possible therapies in many pathologies, such as benign prostatic hyperplasia, prostate cancer, hypogonadism, stress urinary incontinence, osteoporosis, cancer-related cachexia, Duchenne muscular dystrophy, breast cancer, and Alzheimer’s disease.^5-7^ SARMs were also investigated to enhance sexual function and in male contraception.^2^^,^^8-10^ Nevertheless, SARM molecules have still not obtained any marketing authorization in Europe or the United States.^8^^,^^11-13^ Moreover, SARM-containing products are illegally placed on the market with features resembling the aspect of a food supplement (food supplement-like), mainly sold on the internet.^12^^,^^13^

The use of SARMs by athletes or recreational users to enhance athletic performance has been described.^14-16^ Food and Drug Administration warnings and scientific literature report serious life-threatening health problems related to the use of SARMs marketed as dietary supplements, such as increased risk of heart attack or stroke, psychosis/hallucinations, sleep disturbance, sexual dysfunction, liver injury and acute liver failure, infertility, pregnancy miscarriage, and testicular shrinkage.^17-22^ A cross-sectional survey recently pointed out the adverse effects experienced by SARM users as mood swings, decreased testicular size, acne, hair loss, lethargy, irritability, yellow vision, and increased blood pressure.^14^^,^^23^

The World Anti-Doping Agency banned SARMs from competitions as anabolic agents since 2008.^24^^,^^25^ In the last years, the illegal sale of food supplement–like products containing SARMs has spread on the web. The claimed tissue specificity of SARMs led to a broad diffusion of illegal websites selling these products to improve performance in sports, particularly bodybuilding. Moreover, the oral dosage form of these products facilitates their use in comparison to injectable anabolic steroids. A study on social media showed the increased use of SARMs due to the role of influencers in their promotion.^12^^,^^13^ Surveys on the composition of dietary supplements containing SARMs purchased via websites accessible from the United States, the United Kingdom, and Australia were carried out in response to their increasing use by athletes, recreational bodybuilders, and soldiers. Many discrepancies on labeling, the claimed active substance, and the real content were observed.^26-28^

In 2021 the General European Official Medicines Control Laboratory Network, coordinated by the European Directorate for the Quality of Medicines and HealthCare, reported the results of a retrospective and prospective study addressing the occurrence of non-Anatomical Therapeutic Chemical/International Nonproprietary Names (ATC/INN) molecules in suspected illegal or illegally traded products in Europe. The study clearly showed the increasing popularity of SARMs, emphasizing the need of attention and further monitoring of such substances, which are the subject of illegal (internet) trade.^29^ However, research focused on specific geographic markets is limited, leading to a deficiency in understanding the nature and diffusion among users.

This article reports a study supported by chemical analysis on the quality of 13 SARM products bought on websites accessed from Italy. Moreover, an investigation of online search trends with targeted keywords on retail websites and social media was carried out to analyze the interest and exchange of information about these products. This study aimed to answer 2 questions: (1) Is there a growing interest in Italy for these products as conveyed by social media? (2) What is the real content of these illegal products? In other words, do they actually contain what they declare, or are they even falsified? To answer these questions, a study on the Internet search trends on SARMs and on information exchange on socials was performed with qualitative-quantitative chemical analysis of SARM products bought on the Internet. We focused on the Italian market. The aim of this study was to evidence the potential health risks and put the spotlight on the abuse of illegal products to raise consumer awareness.

## Methods

### Websites and product sampling

SARM samples were purchased on the Internet in 2022 on the basis of products available to Italy-based consumers. Products were sourced from suppliers via European Union–based URLs. To select vendors, online retail websites were searched by the Google search engine (Italian terms of service version 5, January 2022), and keywords included the Italian terms *acquista SARMs* (buy SARMs) and *vendita SARMs* (SARMs sale), as well as *SARMs shop* to extend the search to websites reporting the sale information in English.

All the keywords mostly led to the same websites. Among these, 9 websites were pursued that had the *.eu* country name in the domain and offered a variety of SARMs. The provenances (IP addresses) of the retail websites were investigated by the site https://www.ip-adress.com/. Two websites were selected for product purchase. Specifically, we prioritized vendors located in Europe (as requested by the Italian Regulatory Authority) who advertised muscle-building effect, allowed prepaid credit card transactions, offered the most common SARMs (as emerged from the social forums and scientific literature),^26^^,^^28^ and sold SARMs whose reference standards were commercially available.

In parallel, relevant free access forums, Facebook groups (https://www.facebook.com/groups/sarms.peptides.nootropics/ and https://www.facebook.com/groups/193391555902988/), Telegram channels (https://t.me/suppsit), and thematic Reddit forums (https://www.reddit.com/r/SARMs/s/Uh369ZpHU0) accessible from Italy were followed to identify the most popular products and undertake digital observation of aspects related to the increasing interest for these molecules. In practice, 1 researcher from our group accessed the aforementioned platforms and passively observed posts and discussions without actively engaging with the participants. The researcher used the terms *SARMs*, *stacking*, *muscle*, and *booster* as well as those used in the Google search. The information exchanges among users were instantaneous observations caught with screenshots; all identifiable characteristics were removed and data anonymized.^30^

**Figure 1:**
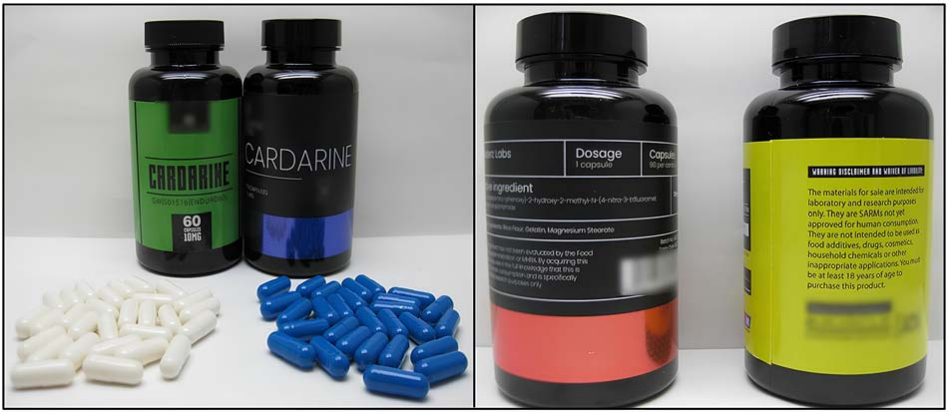
Examples of photographs of SARMs products.

Overall, the study of websites led to the selection of 13 food supplement–like products claiming to contain SARMs. Samples from 2 selected websites were purchased by the Italian Medicines Agency disguised as a private buyer. Upon arrival, samples were given unique identifiers and examined to ensure integrity. All the samples arrived intact and sealed and were stored at room temperature prior to analysis. Each sample was photographed, and the packaging and label were visually inspected to evidence misleading information. Figure 1 provides photographic images of the samples.

The study was approved by the director of the National Center for the Control and Evaluation of Medicines and by the director of its chemical unit.

### Trend of searches

The public interest in SARMs was analyzed by Google Trends (Italian terms of service version 5, January 2022) investigating the search queries “SARMs” for “all categories” for the country “Italy” between March 2013 and February 2023. Google Trends is an online tool that allows users to graph the frequency of searches for single or multiple terms.^31^^,^^32^

The obtained graphs are normalized on a relative basis and can display defined time intervals or geographic areas. By using the Trends Explore tool of Google Trends, it is possible to obtain a graph showing the curve of the popularity of a term over time in comparison with other searches.^31^ All results returned by Google Trends are expressed as relative normalized search volume numbers, with sets of data divided by a common variable to cancel out its effect on the data, and are presented on a scale from 0 to 100, where 100 represents a maximum search interest at a given time and place. The numbers reflect how many searches have been performed for a particular term, relative to the total number of searches performed with Google over time. Each point on the graph is divided by the highest point, which is conventionally set at 100.^31-33^

### Mass spectrometry

The identification analysis was performed on a liquid chromatograph (1290; Agilent Technologies) mass spectrometer quadrupole time-of-flight (6520; Agilent Technologies) apparatus working with a dual electrospray ionization source in positive and negative mode. Data were recorded with MassHunter Data Acquisition version B.06.01 and processed with MassHunter Qualitative Analysis software version B.07.00 (Agilent). All solvents and reagents were liquid chromatograph mass spectrometer grade (Merck KGaA). SARM and no-SARM reference standards were purchased from LGC Limited, Merck, Santa Cruz Biotechnology, and the European Directorate for the Quality of Medicines and HealthCare.

For each product, the powder contained in a capsule was dissolved in 100 mL of methanol. The solution was sonicated for 10 minutes and then magnetically stirred for 1 hour. The solution was then diluted 1:10 in acetonitrile/water (1:1 v/v) containing 0.1% formic acid and filtered on 0.22-μm polytetrafluoroethylene filters. The high resolution of the instrument permitted accurate measurement of the ionic mass in mass spectrometry mode and calculation of the chemical formula of the unknown molecules in the samples. Target tandem mass spectrometry mode revealed the fragmentation pattern of the molecule and its identification by comparison with a reference standard. Fragmentation patterns were obtained at 20 and 40 V of collision energy offset. Auto tandem mass spectrometry analysis was useful to identify pharmaceutical active substances by the Agilent MassHunter Forensics and Toxicology Personal Compound Database and Library (B.07.01), but any SARM’s spectrum was included in the commercial database.

Mass spectrometer parameters were as follows: fragmentor, 100 V; nitrogen, 300 °C; drying gas, 10 L/min; nebulizer, 40 psig; and capillary voltage, 4000 V. Chromatographic separation was carried out on a Zorbax Extend-C18 (2.1 × 50 mm) 1.8-μm particle size column with a linear gradient elution from a high concentration of water (95:5 water/acetonitrile containing 0.1% formic acid) to a high concentration of organic solvent (5:95 water/acetonitrile containing 0.1% formic acid) in 15 minutes. The flow rate was set at 0.4 mL/min and the injection volume was 1 μL. The column temperature was 35 °C and the autosampler was thermostated at 10 °C.

### Quantitative nuclear magnetic resonance

Quantitative analysis on the capsules containing SARMs was performed by nuclear magnetic resonance (NMR). Quantitative NMR (qNMR) analysis establishes the assay of the active ingredient from the area of the specific signal of the analyte in the spectrum, as compared with the area of a reference standard of known quantity. The analysis was performed with a Bruker Avance NEO spectrometer (Bruker BioSpin Gmbh) operating with a field of 14.09 T and at the temperature of 298 K. All spectra were recorded with Bruker TopSpin version 4.1.3 and processed with IconNMR software (Bruker BioSpin).

Due to the presence of fluorine in the structures of SARMs, the ^19^F isotope was used as the resonance nucleus to achieve high specificity, as no elements other than fluorine are detectable in the operating conditions. The ^19^F qNMR method was validated as a screening method (method description and validation are reported elsewhere).^34^

Materials were as follows: dimethylsulfoxide (DMSO; Sigma-Aldrich), deuterated dimethylsulfoxide (DMSO-d6; Cambridge Isotope Laboratories), and 2,4-dichlorobenzotrifluoride as the certified reference standard for NMR quantification (Sigma-Aldrich).

For each analysis, 100 mg of capsule content was dissolved in DMSO (1.3 mL); after mixing, the insoluble matter was discharged, and an accurate amount of the liquid phase was transferred into a vial. A precise amount of a reference standard solution in DMSO-d6 (10 mg/mL) was added to the vial, and the solution was transferred to an NMR tube for spectra acquisition.

The ^19^F NMR spectra showed 2 or 3 signals depending of the number of fluorine atoms in the SARM structure, and these signals had acceptable resolution for quantification.

## Results

### Retail website search and social media

Of the 9 observed retail websites, 7 indicated that SARM products were “for research only” or “for research purposes”; however, in all cases, information was reported on dosages and phases of the bodybuilding plan (ie, in the bulking or cutting phase). Discordant claims such as “take the recommended dose,” “dosage for research only 18+,” and “it does not replace a balanced diet” were found on the same websites. To give credibility to the websites, links to scientific literature sites (PubMed, PubChem, National Institutes of Health) or Wikipedia were sometimes included in the web page. In 1 retail website, a false illegible certification was found attributed to European Medicines Agency good manufacturing practices.

The IP addresses of 6 retail sites evidenced that the apparent locations of the websites, as reported on their web pages, were often different from the real ones, as obtained by https://www.ip-adress.com/. For instance, 2 websites claimed to sell from Italy, but the IP address analysis indicated that they were located in Finland and Canada. In 3 cases, the country of the retail website was not specified on the web page. This was expected as many illegal websites use systems to disguise the real location of the site.^35^

Following social media to obtain information on the products, we observed 3 important aspects:

• The exchange of information among SARM users: new users require information from expert users about means of administration and “stacking” with other products via free forums, Facebook, Telegram, and thematic Reddit forums, where anonymity is possible.

• The sale of products containing SARMs also takes place directly on private channels (Telegram).

• The claim “for research use only” for products containing SARMs does not hamper their sale to the general public presumably not involved in research activities, such as bodybuilders.

An example of this last observation is presented in the sentences in Box 1.

Box 1
Sample sentences on SARMs found in a free access forum and translated into English (originally Italian).

*Although Cardarine has not been approved by the US Food and Drug Administration (FDA), users say it is effective. Many say it helped them lose weight and tone their muscles.*

*The potential applications of Stenabolic are varied and interesting, making it a fascinating pursuit. People do not know and involve exactly its safe uses.*

*While there is no hiding the fact that Ostarine side effects, such as heart attacks and liver damage, can be serious, these side effects only affect a small number of people. Furthermore, this drug effectively increases muscle and reduces fat, testosterone and stamina.*

*The ibutamoren on our list seems to be the most interesting option for those new to the best SARMs. From a chemical point of view, it does not present serious risks for those who use it, but some risks exist.*

*Some companies claim to offer the best results, others may cause health problems. For this reason, it is advisable to do some research before taking them!*

Particularly relevant in the spread of SARMs through bodybuilding forums is the role of the “bro”—that is, an expert bodybuilder giving instructions about tactics for the training, the best products and dosages to take, and the stacking with other products. The sharing of anecdotes and advice, presented as facts but without scientific basis, among expert and beginner users is called “broscience.”^36-38^ Some sentences caught among users on websites are noted in Box 2.

Box 2
Example of a conversation between a “bro” and a beginner user caught in a thematic free access forum and translated into English (originally Italian).

Example of post:

*Only a few SARMs have been tested in human clinical trials even though they have been under study for several years, many say it is the fault of the pharmaceutical companies who do not want them to be sold.*

*I attach general studies and more complete explanations:*

https://www.ncbi.nlm.nih.gov/pmc/articles/PMC2907129/

https://lupinepublishers.com/reproducti...000103.php.

Example of conversation:

*USER 1: I did a 12 week stack of LGD at 10 mg + S4 at 75 mg + RAD140 at 20 mg.*

*I had as a result a great increase in strength, approximately 25 kg ..., (eg, if I previously trained on the bench with 85 kg with the stack I train with 110 kg). In terms of mass I gained around 9\10 kg in 12 weeks.*

*USER 2: WOW 10 kg in 12 weeks, practically growing quickly day after day* 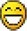.

*Have you tried ostarine alone?*

*And as a PCT, what do you recommend that is easily available?*

*USER 1: ahhah, yes* 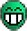 *4 years ago I was anorexic and I weighed 48 kg now I weigh 80* 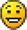 *and I’m, 1.71 height, obviously it wasn’t all mass, a bit of fat, a bit of bone density, joints and glycogen in the muscles to give the 10 kg more but these are concepts that are a bit more from a gym forum haha.*

*Yes I’ve tried it, it doesn’t give you much, but it’s excellent for recovering from injuries, also for training...*

*USER 2: WOW* 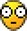 *it’s a huge increase in strength, I knew that SARMs are used a lot in sports to recover and be able to train more. Can you confirm? However they are really interesting as molecules, they will probably be the next step after synthetic steroids.*

### Trend of searches

**Figure 2:**
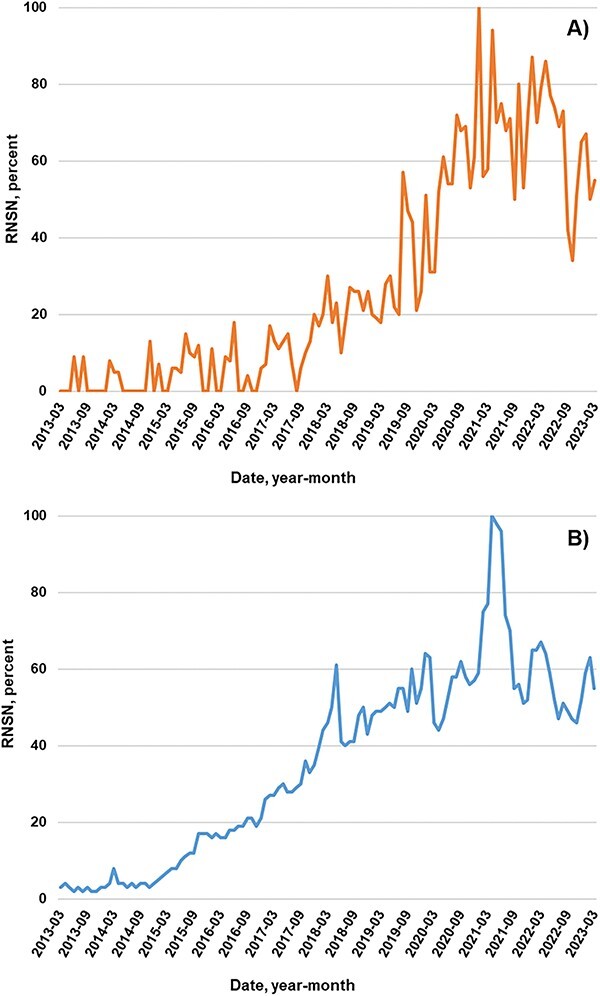
Google Trends search interest of the term SARMs in Italy (Panel A) and the World (Panel B) between 2013 and 2023. Search interests are expressed as relative normalized search volume numbers.

Figure 2A depicts the relative normalized search volume numbers of the Google search in Italy and shows a progressive growth over time starting from approximately 2018 to 2019. Interest was highest at the beginning of 2021, ranging from 90 to 100 (100 is the highest frequency of searches of the term), and then remained mostly stable over the following 2 years, resulting in a striking interest for these products. An increasing trend was also observed for *modulatore selettivo del recettore degli androgeni* (data not shown).

As a comparison, we plotted the Google trend searcher worldwide (Figure 2B) , and the results showed a similar increase in searches, with a peak in the same time range. It is worth mentioning that the interest worldwide appeared earlier, specifically at the end of 2014, as observed in a previous study that explored a different time range.^33^

These data can be correctly interpreted considering that Google Trends is an application to measure search interest in a particular topic, in a particular location, and within a specific time range. Google Trends data are pulled from a random unbiased sample of Google searches. In the graph, 100 is the maximum search interest for the time and location selected. The percentage of searches for a specific topic is calculated as a proportion of all searches at that time and location.^31^

Considering that the trends obtained are a comparison with all searches performed with the same search engine, the increase of interest for these substances is evident.

## Results

### Visual analysis

Table 1 presents the results of the visual inspection of the samples. Labels of all products report statements such as “for research purposes” or “not for human consumption”; in spite of this, the labels also state “supplement facts” with a “serving size” or “dosage,” which allude to human consumption. All the information required for legal products intended for human consumption are untrackable, imprecise, or missing. Samples from supplier A report the name and address of the manufacturer. In all samples from supplier B, the address of the manufacturer and the country of manufacture are absent, but a brand name is available on the label. It is not clear if the brand name corresponds to the manufacturer or the importer. All samples from supplier B show the same batch number and the same expiry date (even if the active substance is different). In a number of samples from supplier A, the same batch numbers and expiry dates are repeated. In all samples, no instruction is available on storage conditions or the mode to take the product. No information on possible interactions with other medicines is available on labeling. Age restrictions are indicated only in products from supplier A; moreover, the reported daily dosage for the same active substance is different between the suppliers, indicating that no scientific studies support the recommended dose. Other ingredients stated on the label are gelatine, rice flour, cellulose microcrystalline, and magnesium stearate (Table 1), and despite the blue shells observed for the majority of samples, no colorants were indicated on the packaging.

**Table 1:** Results of the visual inspection on SARM samples.

	**Labeled**			**Reported manufacturer**		**Labeled**	
**Sample No.**	**SARM**	**Dosage for capsule and serving size**	**Batch and expiry date**	**Name/brand**	**Country**	**Other ingredients**	**Age restrictions**
**Supplier A** ^a^				Yes/yes	Yes	Gelatine, rice flours, microcrystalline celluloses	>18 y
1	Cardarine	10 mg/cps 2 cps	027 10/2025				
2	Andarine	15 mg/cps 2 cps	027 10/2024				
3	Ostarine	15 mg/cps 2 cps	0222 10/2025				
4	Ligandrol	8 mg/cps 2 cps	027 10/2025				
5	S-23^b^	15 mg/cps 2 cps	022 8/2024				
6	Ibutamoren	10 mg/cps 2 cps	023 10/2025				
7	YK11^c^	10 mg/cps 2 cps	022 8/2024				
**Supplier B** ^d^				No/yes	No	Rice flour, gelatin, magnesium stearate	None
8	Cardarine	10 mg/cps 1 cps	042022 4/25				
9	Andarine	25 mg/cps 1 cps	042022 4/25				
10	Ostarine	10 mg/cps 1 cps	042022 4/25				
11	Ligandrol	10 mg/cps 1 cps	042022 4/25				
12	S-23^b^	10 mg/cps 1 cps	042022 4/25				
13	Ibutamoren	10 mg/cps 1 cps	042022 4/25				

Abbreviations: IUPAC, International Union of Pure and Applied Chemistry; MHRA, Medicines and Healthcare products Regulatory Agency; SARM, selective androgen receptor modulator.

a Warning disclaimer on the label: supplier A, “The materials for sale are intended for laboratory and research purposes only. They are SARMs not yet approved for human consumption. They are not intended to be used as food additive, drugs, cosmetic, household chemical or other inappropriate applications.” Labeled storage conditions were not reported.

b (2S)-3-(4-chloro-3-fluorophenoxy)-N-[4-cyano-3-(trifluoromethyl)phenyl]-2-hydroxy-2-methylpropanamide (IUPAC name).

c Methyl (2*E*)-2-[(8*R*,9*S*,10*R*,13*S*,14*S*,17*S*)-2′-methoxy-2′,13-dimethyl-3-oxospiro[1,2,6,7,8,9,10,11,12,14,15,16-dodecahydrocyclopenta[a]phenanthrene-17,5′-1,3-dioxolane]-4′-ylidene]acetate (IUPAC name).

d Warning disclaimer on the label: supplier B, “This statement has not been evaluated by the Food and Drug Administration or MHRA. By acquiring this product you are in the full knowledge that this is not for human consumption and is specifically for market/research purposes only.” Labeled storage conditions were not reported.

### Qualitative and quantitative analysis

Regarding label reliability, the results of the qualitative analysis by mass spectrometry (Table 2) evidenced the presence of the stated SARM in about 70% of samples (samples 1, 3, 4, 6, 8-12). In 23% of samples, the declared SARM was replaced by a different SARM (samples 2, 5, and 13). No SARM was detected in sample 7. In 30% of the samples, undeclared pharmaceutical active substances were detected (samples 2, 4, 5, and 7). In particular, selective estrogen receptor modulators (tamoxifen and/or clomifene) were detected in samples 4 and 7; epimethandienone samples 4, 5, and 7; testosterone in sample 2; and tadalafil in sample 5. The copresence of >1 active substance and cross-contaminants was observed in >60% of samples (the last 8 samples of Table 2). In 4 of 13 samples, the declared active substance was absent (the last 4 samples of Table 2).

**Table 2:** Results of the qualitative analysis by mass spectrometry.

**Sample No.**	**Labeled SARM**	**Active substances found**
1	Cardarine	Cardarine
8	Cardarine	Cardarine
3	Ostarine	Ostarine
10	Ostarine	Ostarine
6	Ibutamoren	Ibutamoren
9	Andarine	Andarine Contamination of cardarine
12	S-23^a^	S-23 Contamination of ligandrol, ostarine, and ibutamoren
11	Ligandrol	Ligandrol Contamination of cardarine
4	Ligandrol	Ligandrol Clomifene Tamoxifen Epimethandienone
5	S-23^a^	Contamination of cardarine Epimethandienone Tadalafil
2	Andarine	Cardarine Testosterone
13	Ibutamoren	RAD-140 Contamination of ligandrol
7	YK11^b^	Epimethandienone Contamination of tamoxifen

Abbreviations: IUPAC, International Union of Pure and Applied Chemistry; SARM, selective androgen receptor modulator.

a (2S)-3-(4-chloro-3-fluorophenoxy)-N-[4-cyano-3-(trifluoromethyl)phenyl]-2-hydroxy-2-methylpropanamide (IUPAC name).

b Methyl (2*E*)-2-[(8*R*,9*S*,10*R*,13*S*,14*S*,17*S*)-2′-methoxy-2′,13-dimethyl-3-oxospiro[1,2,6,7,8,9,10,11,12,14,15,16-dodecahydrocyclopenta[a]phenanthrene-17,5′-1,3-dioxolane]-4′-ylidene]acetate (IUPAC name).

Quantitative information obtained from ^19^F qNMR analysis provided the content of the main active substance in the 10 samples containing fluorine in the chemical structure.

**Figure 3:**
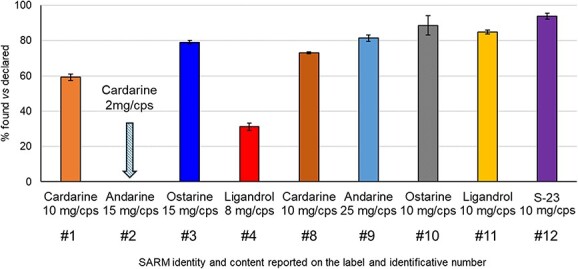
Content in % of the active ingredient of the quantified SARMs. Sample # 2 was labelled as Andarine 15 mg/cps but it contained 2 mg/cps of Cardarine.

As seen in Figure 3, samples 9 to 12 were close to the declared content (80%-95%); samples 1, 3, and 8 were in the range of approximately 60% to 80% of labeled content; and sample 4 was highly underdosed. Sample 2 contained approximately 2 mg/cps of cardarine instead of the declared 15 mg/cps of andarine, and sample 5 contained cardarine in traces instead of the labeled S-23 (not reported in Figure 3). In total, cardarine was present in 6 samples at levels ranging from few milligrams per capsule (not quantified) to 70% of the declared content, but it was declared in only 2 samples.

## Discussion

The results on retail websites evidenced the implicit indication for human consumption of SARM products, demonstrating that the statement “for research only” was not truthful with respect to the real scope of sale. The increasing interest for these products, as revealed by the increasing trend of searches on the web, needs higher attention to the fraudulent claims of these products, which are not approved as medicines or as ingredients in food supplements. The study of trends of interest on specific terms is more and more diffused in many fields,^39^ and this tool has been specifically applied to SARMs.^33^^,^^40^ In the present study, we evidenced the increased interest of SARMs in Italy, which started later in comparison with the entire world and showed a near plateau in the last 2 years.

Internet forums are a good means to obtain cultural findings, and the interest of the medical community for this instrument is rising.^41^^,^^42^ Although the observation of forums and websites was aimed at obtaining information on the most used products and not to carry out a systematic study on social media, some information emerged that was useful for the purpose of the search.

The discussions on specific forums and the claims on the retail websites induce the consumption of these products, and it is evident that the unapproved status of SARMs as human medicines or food supplements is not a deterrent for purchase and consequent consumption. The abuse of dangerous products is encouraged by the influence of consumer decision making by the word-of-mouth extolled by social media as well as the emerging phenomenon of “broscience.”^12^^,^^13^^,^^37^^,^^43^ On the studied social media, there were no discordant voices clearly indicating the risks to consume substances not yet authorized. This fact raises the urgency to improve the efforts to inform the target population on the risks to consume illegal substances whose health effects were not yet deeply investigated.

The visual analysis of the samples highlights the contradictory information of the labels. Moreover, the lack of information about the manufacturer name and address in the samples from supplier B does not permit legal action in case of severe adverse reactions due to the product. For supplier A, we could not assess if the manufacturer name and address on the label were true, because of the difficulties to obtain information from the authorities of a country outside the European Union (Georgia). The repetition of the batch numbers and expiry dates in different samples suggests that these numbers are not truthful or based on stability studies. Another point worth of discussion is the presence of other ingredients in the products, such as gelatine, rice flour, and cellulose microcrystalline. The quality of these excipients may be affected by the substandard manufacture characterizing illegal or unauthorized products. For instance, rice is a staple crop naturally capable of accumulating potentially toxic trace elements such as cadmium or arsenic.^44-46^ Gelatin is a natural soluble protein obtained by partial hydrolysis of the collagen produced from the bones, hides, and skins of animals.^47^^,^^48^ The European Medicines Agency advised on minimizing the risk of transmitting animal spongiform encephalopathy agents via a product of animal origin, such as gelatine,^49^ addressing specific consideration to the origin of the material and the production processes. In illegal products, there is no evidence that such considerations have been properly addressed. Typically, the body of the opaque hard capsule shell is made of gelatine with other components, such as preservatives or colorants that are normally authorized as food-grade additives. The absence of any colorant among the list of excipients, despite the blue color of the capsules, aroused suspicion about the presence of other ingredients of unknown quality besides those noted in the formulation. In addition, the presence of identical excipients in all samples of supplier A and the same excipients in all samples of supplier B make us suppose the absence of formulation studies to optimize the release of each SARM and to minimize the side effects and degradation of the product.

Qualitative/quantitative results point to very low quality of the analyzed samples, suggesting that the health risk for the users is related not only to the intake of illegal and unauthorized substances but also to the consumption of an uncontrolled mix of different active ingredients. The copresence of various pharmaceutical substances (SARMs, androgen steroids, selective estrogen receptor modulators, phosphodiesterase 5 inhibitors) that can interact could have an unknown impact on health. It should be underlined that the combination of different SARMs or SARMs and other active pharmaceutical ingredients has not been examined in clinical trials, so no toxicologic data are available for this kind of product containing >1 active ingredient. The presence of contaminants from other active substances clearly indicates the absence of good manufacturing practices in the manufacturing process and lets us presume the biological and chemical unsafety of the products. The nonuniform quantitative results among the samples, even among those containing the same drug substance, represent a further threat to users exposed to the risk of taking an amount of active substance significantly different from time to time. Moreover, the SARM dosages used in clinical trials were generally less than those recommended in these products.^50^^,^^51^

## Conclusion

The results obtained in this study, despite the limited number of samples from a few retail websites found in the clear web, highlighted that SARMs deceptively sold as food supplements or similar products are a health hazard, not just for containing illegal unapproved active substances, but also for containing undeclared active substances.

Moreover, the present study highlighted the increased interest of SARMs on the web in Italy and the presence of an unregulated web market, with misleading claims on the web pages and on the labeling of SARM products. Furthermore, this investigation evidenced that the exchange of information on these products passes through social networks and advice of “expert” users.

In Italy, websites selling illegal products are routinely banned by specific police forces, but other illegal retail websites are promptly reopened. The aim of the present study was to increase the attention of health authorities and health professionals on this illegal market to alert target groups about the risks of consuming unapproved products.
